# Soft tissue protection from exposed K-wires

**DOI:** 10.1308/003588412X13373405385214u

**Published:** 2012-07

**Authors:** A Cheung

**Affiliations:** West Hertfordshire Hospitals NHS Trust,UK

**Figure 1:**
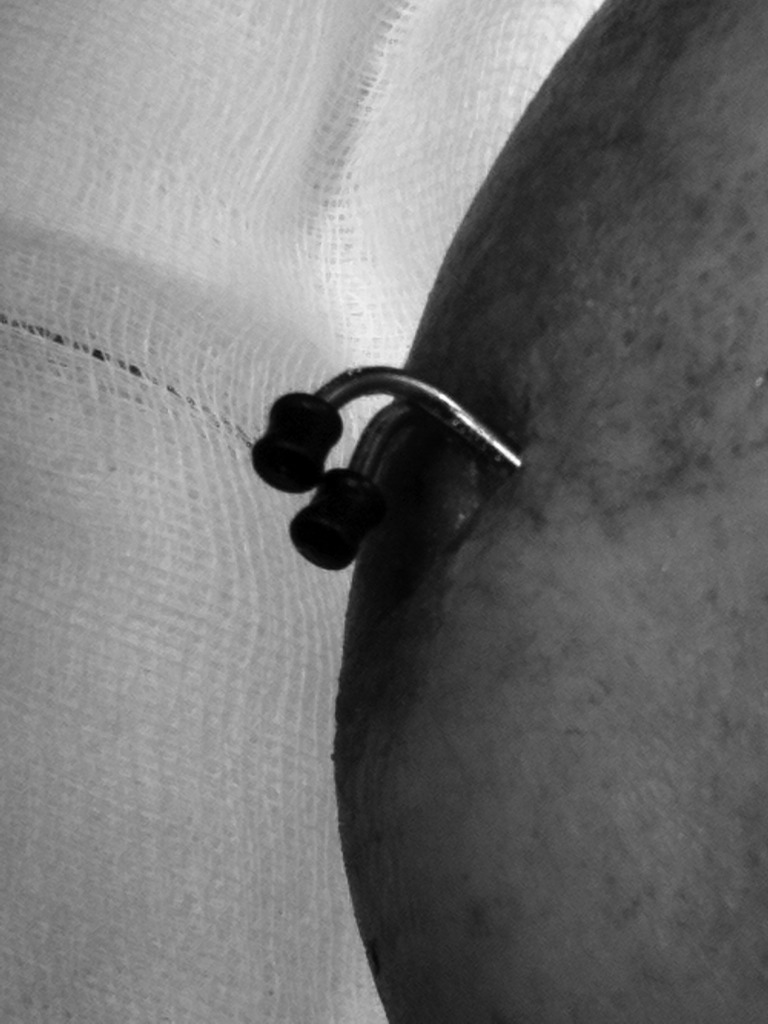
Exposed K-wires covered with a syringe gasket

K-wires may be used to maintain fracture reduction for several weeks in orthopaedic surgery. Exposed sharp ends are a potential risk to the surgeon and patient. Covering the exposed wire end with a 1ml syringe gasket (black bung located at plunger tip) provides secure protection (Fig 1). This is a cheap and effective method of preventing drape perforation and soft tissue injury.

